# Campbell and Cochrane evidence on promoting cognitive capacity across life course: a mapping review

**DOI:** 10.1093/ageing/afaf306

**Published:** 2025-10-26

**Authors:** Vivian A Welch, Monserrat Conde, Victoria I Barbeau, Elizabeth Ghogomu, Shawn Kuria, Hind Sabri, Yanfei Li, Kathy Tong, Ali S Abud, Keshvi Vithlani, Vanessa De Rubeis, Chandni M Jacob, Ritu Sadana

**Affiliations:** School of Epidemiology and Public Health, University of Ottawa, Ottawa, Ontario, Canada; Bruyère Health Research Institute, Ottawa, Ontario, Canada; Nuffield Department of Primary Care Health Sciences, University of Oxford, Oxford, United Kingdom; Bruyère Health Research Institute, Ottawa, Ontario, Canada; School of Epidemiology and Public Health, University of Ottawa, Ottawa, Ontario, Canada; Bruyère Health Research Institute, Ottawa, Ontario, Canada; Bruyère Health Research Institute, Ottawa, Ontario, Canada; Bruyère Health Research Institute, Ottawa, Ontario, Canada; Bruyère Health Research Institute, Ottawa, Ontario, Canada; Lanzhou University, Lanzhou, Gansu, China; Bruyère Health Research Institute, Ottawa, Ontario, Canada; Bruyère Health Research Institute, Ottawa, Ontario, Canada; Bruyère Health Research Institute, Ottawa, Ontario, Canada; Division of Universal Health Coverage – Life Course, World Health Organization - Maternal, Newborn, Child, Adolescent Health and Ageing, Geneva, GE, Switzerland; Division of Universal Health Coverage – Life Course, World Health Organization - Maternal, Newborn, Child, Adolescent Health and Ageing, Geneva, GE, Switzerland; Division of Universal Health Coverage – Life Course, World Health Organization - Maternal, Newborn, Child, Adolescent Health and Ageing, Geneva, GE, Switzerland

**Keywords:** cognition, ageing, equity, reviews, life course, older people

## Abstract

**Background:**

Cognitive capacity and function affect daily activities, independence, and overall well-being across the life course.

**Objective:**

To map and synthesise evidence on interventions that measured cognitive capacity at any life stage across the life course from Cochrane and Campbell systematic reviews.

**Design:**

Mapping review.

**Methods:**

The Cochrane and Campbell libraries were searched up to 1 May 2024 for systematic reviews of interventions that measured cognitive capacity across all ages. Data on interventions and outcomes were coded using the International Classification of Function and the International Classification of Health Interventions. We coded for equity characteristics using PROGRESS-Plus. Methodological quality was assessed with AMSTAR2.

**Results:**

We included 34 Campbell and 31 Cochrane reviews, with over half (55%) rated as high quality. Most reviews (80%) included studies from high-income countries, with only 12% including studies from low-income countries. Of the 30 reviews that planned a subgroup analysis across equity characteristics, only eight did so. Most reviews included multiple age groups (63%), but none evaluated differences in cognitive outcomes across more than two age categories. Practical support interventions (60%) and intellectual function outcomes (51%) were most common; however, the interventions and outcomes varied at different life stages, reflecting a focus on development in younger ages and on maintaining cognitive function or prevention of decline in older ages.

**Conclusion:**

This work highlights the need for a comprehensive life course approach to cognitive interventions, incorporating equity considerations and age-appropriate outcome measures.

## Key Points

- Systematic reviews synthesise and critically appraise all available evidence on the effectiveness of specific interventions.
- Most Cochrane and Campbell reviews on cognition examine practical support interventions and measures of intellectual function.
- The mapping showed that the interventions and outcomes studied varied across the different life stages.
- Outcomes and interventions should be tailored to reflect the specific needs and characteristics of each life stage.

## Background

The life course perspective is a person-centred approach which focuses on how exposure to psychosocial, biological, and environmental factors across the life course influences an individual’s health and well-being. It considers the interaction of multiple protective and risk factors over time, emphasising key developmental stages, such as newborn, early childhood, and adolescence and women’s experiences during and pre-pregnancy, which are pivotal for shaping long-term health outcomes [1, 2]. The approach spans from childhood through adolescent and adult health, to healthy ageing and dignified end-of-life care [3].

Healthy ageing is viewed as a continuous process that requires optimising health trajectories throughout life rather than focusing solely on interventions later in life [4]. Promoting good health in earlier stages is crucial for achieving well-being in later years. It aligns with the World Health Organisation (WHO) and United Nations’ Decade of Healthy Ageing, which promotes well-being, positive health attributes beyond medical conditions and disabilities, and taking a life course approach that provides accumulative benefits for older people and the next generation [1, 2].

Societal factors such as neighbourhood socioeconomic deprivation are also crucial in shaping health across life course. Furthermore, people experience different opportunities and barriers based on sex, gender, and intersecting identity factors, which may lead to health inequities that shape trajectories of health across the life course [5].

The WHO defines intrinsic capacity of older people as the composite of all physical and mental capacities, including cognition, locomotion, sensory, vitality and psychological domains [1]. For people of all ages, cognitive capacity, the ability to perform a range of mental or cognitive functions, is one of the most measured domains of intrinsic capacity [6]. Cognitive function, which encompasses memory, attention, learning, decision-making, and language, is a key aspect of health influencing many abilities across life [1, 7]. It is also closely linked to sleep, as adequate sleep is essential for memory consolidation [8]. Cognitive function is measured in different ways across various ages and stages of life; for instance, outcome measures in young children may assess development, while in older adults, there is a focus on response time and memory. Classification frameworks such as the International Classification of Functioning, Disability and Health (ICF), International Classification of Diseases, and approaches to measure different categories of illness and health, such as the International Resident Assessment Instrument have been used to assess these different dimensions of cognition [9–11]. The ICF framework, in particular, uses a comprehensive approach to map cognitive function and its impact on daily activities, participation, and well-being across the life course, focusing on function rather than disease [12].

Interventions to improve cognitive function differ substantially with age. For example, ​​nutritional supplements to promote growth and development in children, and cognitive training for memory in older adults [13, 14]. Improving cognitive function at any life stage is thought to improve lifelong cognitive capacity [2]. It is therefore important for decision-makers to know the effectiveness of interventions and outcome measures used to evaluate cognition at all stages.

Systematic reviews comprehensively synthesise all available studies on a particular question and critically appraise the evidence [15]. For this reason, they are valued for helping inform decision-making and setting priorities for future research.

This mapping review uses a life course approach to describe systematic reviews of interventions that measure cognition at different life stages. The findings can be used to identify research gaps and inform future research design on cognition across the life course.

## Objectives

The objectives were to:

- Describe interventions that measure cognitive capacity at any point in the life course from systematic reviews.
- Evaluate the description of sex, gender, intersecting identity factors and the global representation of studies included in the systematic reviews.
- Map interventions and outcomes across the International Classification of Health Interventions (ICHI) and ICF frameworks.

## Methods

We followed rapid review guidance and an evidence and gap map approach to document the interventions and outcomes assessed for cognitive capacity across the life course [16, 17]. We operationalised cognitive capacity assessment as measures of cognitive function. This manuscript follows the Preferred Reporting Items for Systematic Reviews and Meta-analysis —Extension for Scoping Reviews (PRISMA-ScR) guideline (Appendix 1). The protocol was registered on Open Science Framework (OSF) [18].

### Eligibility criteria

Population: People of all ages and settings were included. People with pre-existing cognitive impairment conditions (e.g. dementia) or chronic conditions who are experiencing cognitive decline (e.g. end-stage renal failure) were excluded.

Interventions: We included preventive, health-promoting, and management interventions that aim to improve cognitive function and capacity (e.g. nutritional supplements, skill building). Pharmacologic and surgical interventions were excluded as they may target specific diseases or conditions.

Comparison: Any comparison was eligible.

Outcomes: Reviews had to assess cognitive capacity or function as a primary or secondary outcome. Cognitive capacity is one of the components of body function in the ICF framework and includes global mental functions, including orientation, intellectual functions, and global psychosocial functions, as well as specific mental functions, including speech, memory, psychomotor functions, and attention [6]. We also included reviews if they measured sleep, mental functions of language, and calculation functions because these outcomes are associated with cognitive function.

Study design: We included Cochrane and Campbell reviews assessing intervention effectiveness or efficacy. Evidence and gap maps, scoping reviews, overviews of reviews, and reviews of prognostic or risk factors were excluded.

### Search strategy

For feasibility, we focused on the Cochrane and Campbell libraries, which are known for high-quality and rigorous systematic reviews and are used to inform policy, clinical decisions, and guidelines.

The Population, Intervention, Comparison, and Outcome (PICO) search tool in the Cochrane Library was used to identify all reviews published before 14 May 2024 and tagged with an outcome under the following categories: cognitive function or findings related to sleep [19]. Web of Science was used to identify all studies published in Campbell Systematic Reviews from 1 January 2018 until 1 May 2024. All Campbell studies published before 2018 were collected from the Wiley Online Library.

### Screening and study selection

Titles and abstracts were screened in duplicate by six reviewers (E.T.G., H.S., K.T., S.K., V.B., Y.L.) using Covidence, with discrepancies resolved through discussion. Relevant full texts were screened in duplicate by five reviewers (H.S., K.T., S.K., V.B., Y.L.) using Covidence, with discrepancies resolved through discussion [20].

### Data collection

Participant characteristics were coded using the PROGRESS-Plus framework (Place of residence, race/ethnicity/culture/language, occupation, gender/sex, religion, education, socioeconomic status, social capital) [21]. The Plus represents other characteristics associated with discrimination and barriers to health, including age, disability, and sexual orientation. Health conditions and comorbidities were also coded.

Age was collected using six categories across the life course, chosen in consultation with WHO: 1. Preconception and perinatal (<1 year), 2. Children under 5, 3. Children 5–9, 4. Adolescents 10–19, 5. Adults 20–60, and 6. Adults over 60. For studies that include more than one life stage, we coded disaggregated data if available in the study. If not, we coded each relevant life stage included.

Assessed outcomes were classified using 10 ICF categories: orientation, intellectual functions, global psychosocial functions, sleep, attention, memory, psychomotor functions, mental functions of language, calculation functions, and fluency and rhythm of speech.

Interventions were classified using eight ICHI categories: assisting and leading exercise for global psychosocial functions, training of global psychosocial functions, education about global psychosocial functions, advising about global psychosocial functions, counselling for global psychosocial functions, practical support with global psychosocial functions, emotional support for global psychosocial functions, and provision of peer support for global psychosocial functions [22] (Appendix 2—definitions and item codes).

Intervention reporting was described using the Template for Intervention Description and Replication (TIDieR) framework [23]. We also documented the type(s) of comparison groups.

### Critical appraisal

For Campbell reviews published in November 2022 or earlier, we used AMSTAR2 ratings from two previous projects in which appraisal was done in duplicate [24, 25]. The methodological quality of the remaining reviews was assessed in duplicate by seven reviewers (A.A, E.T.G., H.S., K.T., S.K., V.B., Y.L.) using AMSTAR2, with discrepancies resolved through discussion (Appendix 3) [26].

### Health equity analysis

We documented whether the reviews planned to evaluate differences in effects across PROGRESS-Plus factors and whether these analyses were carried out. We also assessed if and how the reviews evaluated applicability across PROGRESS-Plus.

### Synthesis approach

Data were summarised in tables and figures to describe the study characteristics, population across PROGRESS-Plus factors, and geographic distribution. Matrices were also used to map interventions and outcomes across the life course.

## Results

### Search results

**Figure 1:**
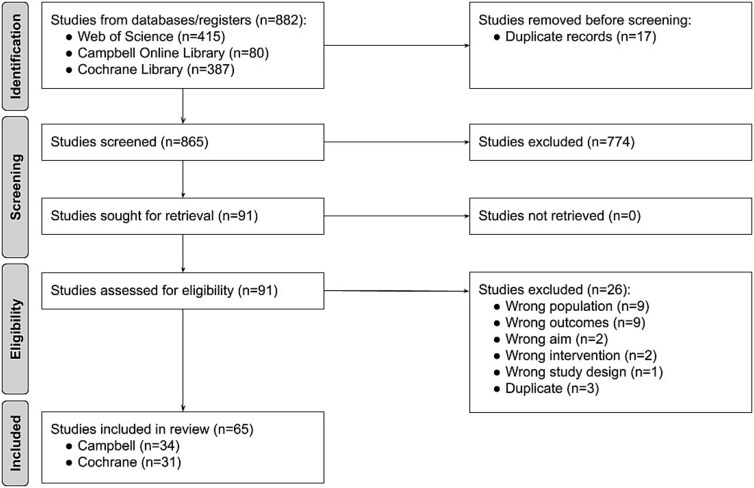
PRISMA flow diagram.

We retrieved 882 records from the library searches and screened 865 after deduplication. Of these records, we excluded 774 during title and abstract screening, and 26 during full-text screening, leaving 65 eligible reviews (Figure 1, Appendix 4—characteristics of excluded studies).

### Description of included reviews

Sixty-five reviews were included [13, 14, 27–89]. Review characteristics are summarised in Table 1 with full datasets available on OSF [90]. The reviews were published between 2003 and 2024 in the Campbell (*n* = 34, 52%) and Cochrane (*n* = 31, 48%) journals. Three reviews were published in both journals but were classified only under the journal where they were published first [52, 53, 60]. Most reviews were funded by university institutions (*n* = 33, 51% reviews) or governments (*n* = 28, 43% reviews).

**Table 1:** Characteristics of included reviews (*n* = 65)

**Characteristics**	**n (%) of reviews/studies**
Publication year	
2000–2005	2 (3%)
2006–2010	4 (6%)
2011–2015	10 (15%)
2016–2020	33 (51%)
2021–2024	16 (25%)
Journal	
Campbell	34 (52%)
Cochrane	31 (48%)
Most common funding sources^a^	
University institutions	33 (51%)
Government	28 (43%)
Non-profit organisations	10 (15%)
Professional associations	8 (12%)
Hospitals	8 (12%)
Sample size (number of included studies)	
Range	0–607 studies
Median	16
Interquartile range	38
Most common locations for the conduct of studies included in the reviews^a^	
USA	42 (65%)
UK	29 (45%)
Canada	22 (34%)
Australia	20 (31%)
Netherlands	18 (28%)
Participant age groups^a^	
Preconception and perinatal	13 (20%)
Children under 5 years	17 (26%)
Children 5–9 years	25 (38%)
Adolescents 10–19 years	34 (52%)
Adults 20–60 years	22 (34%)
Older adults >60 years	15 (23%)
Multiple age groups	41 (63%)
Unspecified	1 (2%)
Reported population sociodemographic characteristics^a^	
Place of residence (urban/rural)	17 (26%)
Race or ethnicity	23 (35%)
Occupation	10 (15%)
Gender or sex	50 (77%)
Religion	1 (2%)
Education	12 (18%)
Socioeconomic status	20 (31%)
Social capital (e.g. marital status)	8 (12%)
Plus factor - age	64 (98%)
Plus factor - disability	8 (12%)
Plus factor - health status (e.g. dementia, disease severity)	14 (22%)
Types of interventions^a^	
Practical support with global psychosocial functions	39 (60%)
Education about global psychosocial functions	17 (26%)
Training of global psychosocial functions	8 (12%)
Assisting and leading exercise for global psychosocial functions	2 (3%)
Counselling for global psychosocial functions	2 (3%)
Emotional support for global psychosocial functions	2 (3%)
Provision of peer support for global psychosocial functions	1 (2%)
Advising about global psychosocial functions	0
Types of outcomes^a^	
Intellectual functions	33 (51%)
Mental functions of language	20 (31%)
Calculation functions	14 (22%)
Psychomotor functions	13 (20%)
Global psychosocial functions	10 (15%)
Memory functions	8 (12%)
Attention functions	6 (9%)
Sleep functions	6 (9%)
Fluency and rhythm of speech functions	4 (6%)
Orientation functions	0
No outcomes	12 (18%)

^*a*^ *Reviews may be counted more than once if they include studies crossing multiple categories*

**Figure 2:**
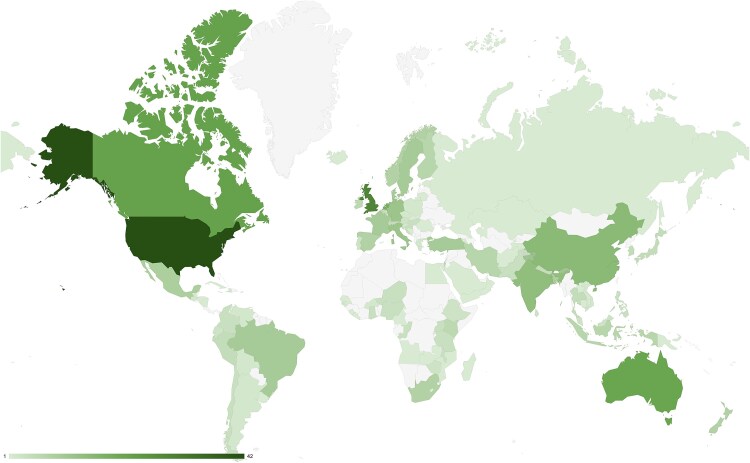
Geographic heatmap of where studies in the systematic reviews were conducted. If a review included multiple studies conducted in the same country, the country was only counted once.

Most reviews had at least one primary study conducted in the USA (*n* = 42, 65%), followed by the UK (*n* = 29, 45%) (Figure 2). Two reviews did not report where studies were conducted [68, 87]. Twenty-nine (45%) reviews included primary studies across multiple economies, and 49 (75%) reviews included studies across multiple regions. The reviews included studies conducted across all four income categories; however, most were in high-income economies (*n* = 54, 83% reviews) (Appendix 5).

Twenty-six reviews included a mixed population of participants with and without pre-existing health conditions affecting cognition. Twenty-five provided disaggregated data, so we only extracted data related to the participants without pre-existing health conditions. The remaining review lacked disaggregated data but was included as it only involved a small proportion of children with learning disabilities (5.9%) [54].

One (2%) review was empty, with no included studies [62]. Eleven reviews planned analyses but did not find studies measuring cognitive outcomes.

All age groups were represented, with adolescents being most frequently included (*n* = 32, 49%). Forty-one reviews (63%) included multiple age groups across the life stage. The included studies in the reviews ranged from 0 to 607 (median = 6, interquartile range = 38).

Sixty (92%) reviews assessed intervention(s) categorised by one ICHI category, and five (8%) reviews assessed interventions from two or more categories. The most common ICHI categories were practical support (*n* = 39, 60%) and education (*n* = 17, 26%). Interventions on advising about global psychosocial functions were not assessed in any reviews.

The most common outcomes measured were intellectual functions (*n* = 33, 51%) and mental functions of language (*n* = 20, 31%). Orientation was not assessed in any reviews.

### Methodological quality of included reviews

Of the included reviews, 36 (55%) were rated as high, 17 (26%) as moderate, 11 (17%) as low and one (1%) as critically low using AMSTAR2.

Most items were adequately reported, especially the reporting of conflicts of interest (*n* = 65, 100%), research questions, and inclusion criteria (*n* = 64, 98%). The most common items affecting the methodological quality were not reporting funding sources of included studies (*n* = 44, 68%) and not assessing the potential impact of risk of bias in individual studies on the results (*n* = 12, 18%) (Appendix 6).

### Interventions across life course

**Figure 3:**
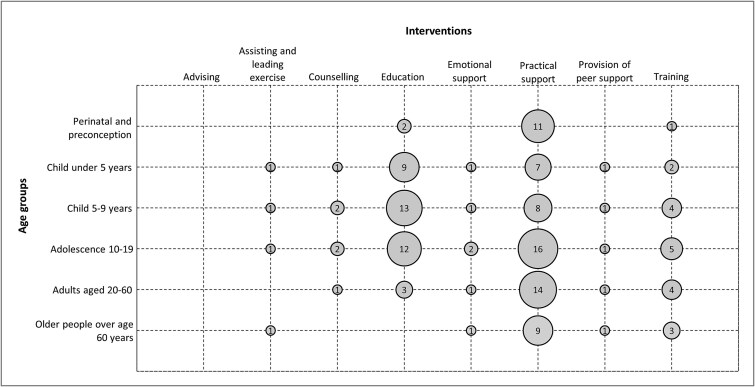
Matrix of reviews as classified by their associated age group(s) and intervention(s). The size of the circles corresponds to the number of reviews (*n* = 65).

Of the 64 reviews that reported participant age, practical support was the most studied, with 7–16 reviews per age group (Figure 3). In the perinatal and preconception age group, 85% of reviews examined practical support, including supplementation.

Education was the second most assessed intervention across the reviews and was most common among studies involving children under 5 (*n* = 9, 53%), children 5–9 (*n* = 13, 52%), and adolescents 10–19 (*n* = 12, 35%) years. These reviews included tutoring, nutrition and lifestyle education, class sizes, preschool, and Montessori interventions. Conversely, education interventions were rarely assessed among reviews of perinatal infants (*n* = 2, 15%), adults (*n* = 3, 13%) or older adults (*n* = 0).

Exercise, counselling, emotional support and peer support interventions had two or fewer reviews across each age group.

When mapping the 35 high-quality reviews, a similar trend could be observed whereby practical support interventions were the most assessed intervention across the entire life course. None of the reviews assessing exercise or peer support interventions were considered high quality (Appendix 7).

### Description of interventions

Of the 12 items from the TIDieR checklist used to assess intervention reporting, only the intervention name and rationale were adequately reported across all 65 reviews. There was no significant correlation between the number of reported TIDieR items and the year of review publication.

The interventions aimed to improve cognitive function (*n* = 56, 86%), prevent cognitive impairment (*n* = 4, 6%), improve sleep outcomes (*n* = 3, 5%), and maintain cognitive function (*n* = 2, 3%). Thirty reviews (46%) described materials and training used. Nineteen reviews (29%) reported the intervention’s provider (e.g. healthcare professionals, caregivers, etc.). Around half of the reviews described how the interventions were delivered (*n* = 34, 52%) and the format (*n* = 32, 49%). The duration and frequencies varied with some interventions being delivered once or twice daily, every other day, weekly, bi-weekly, or monthly, and others only once, 2–3 sessions/week or 10 hours/week. Thirty reviews (46%) reported the settings where the interventions were delivered (e.g. homes, schools, clinical settings, etc.). Eight reviews (12%) described tailoring the interventions, and only one review (1%) described modifying the intervention during the study.

### Outcomes across life course

**Figure 4:**
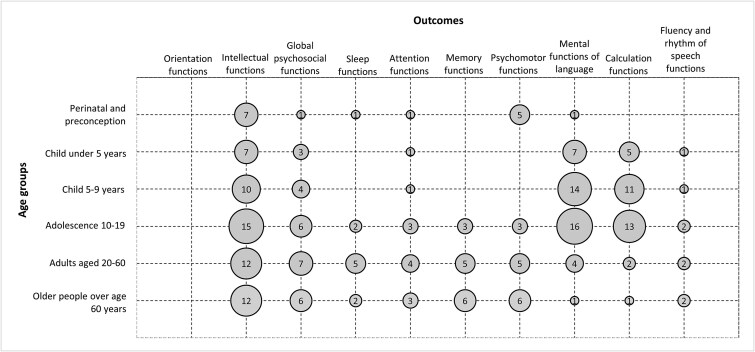
Matrix of reviews as classified by their associated age group(s) and outcome(s). The size of the circles corresponds to the number of reviews (*n* = 65).

Among the 53 reviews with effectiveness data on cognitive outcomes, the most common outcome was intellectual functions (*n* = 33, 62%), with each age group including 7–15 reviews (Figure 4). Measures within this category include intellectual development, overall cognition, overall academic performance, mental development, and executive function.

Mental functions of language (n = 20, 37%) and calculation functions (*n* = 14, 26%) were also commonly assessed in the included reviews. Language outcomes, including literacy, were commonly measured in children under 5 (*n* = 7, 41%), children 5–9 (*n* = 14, 56%), and adolescents 10–19 (*n* = 16, 47%). Conversely, language outcomes were not often assessed among reviews of adults (*n* = 4, 16%) and older adults (*n* = 1, 7%). Calculation outcomes, including math and numeracy test scores, were also commonly measured in children under 5 (*n* = 5, 29%), children 5–9 (*n* = 11, 44%), and adolescents 10–19 (*n* = 13, 38%) years of age but not for adults (*n* = 2, 8%) and older adults (n = 1, 7%).

Sleep, memory, and psychomotor function outcomes were not assessed in children under nine years.

Additional information on the interventions and outcomes across the life course can be found in the appendices (Appendix 8, 9).

### Health equity considerations

#### Participant characteristics across PROGRESS-Plus

The most described characteristics were age (*n* = 64, 98%), gender or sex (*n* = 50, 77%), race/ethnicity (n = 23, 35%), and socioeconomic status (*n* = 20, 31%) (Appendix 10).

#### Equity analysis

Subgroup analyses were planned in 30 (46%) reviews to assess differential effects across PROGRESS-Plus factors, but only eight (12%) had sufficient data to conduct the planned analyses. Among the three reviews that evaluated differential effects across age, two included participants who fell under only one of our age categories [44, 53]. The third review evaluated effects on children 5 years or under compared to children and adolescents 6–18 years of age [55]. No reviews planned to assess differential effects across religion or social capital.

We found no reviews that assessed applicability across PROGRESS-Plus factors for different populations or contexts.

## Discussion

This review aimed to identify and map available evidence from Campbell and Cochrane systematic reviews on interventions that measure cognitive capacity at any life stage. There is a notable gap of studies from low and middle-income countries and the global south in the included reviews. Population health trajectories can vary globally and are influenced by a range of social, environmental, and commercial determinants. These factors can also influence the distribution of cognitive function, with socioeconomic and cultural contexts playing a relevant role in informing adequate tailoring to specific population groups [91]. Future research should enhance the geographical representation of the evidence base and consider approaches to shift the distribution positively.

More than half of the reviews were high-quality, which may reflect the attention to quality standards in both Cochrane and Campbell [92, 93]. However, our quality appraisal also demonstrated that aspects such as reporting funding sources of included studies and assessing the potential impact of risk of bias in individual studies on the results could be improved.

Practical support interventions were the most common across all age groups, but specific interventions were different across the age groups. Although many reviews included two or more age groups, only three conducted a subgroup analysis to examine differences in effects across age. Cognitive capacity is broad, encompassing subdomains that follow different trajectories across the life course, and in each life stage. Subdomains such as vocabulary, which tend to grow during childhood and stabilise with age, vary from others like processing speed, which may decline over time [94]. Interventions and outcomes varied between different age groups, reflecting the developmental stages and functional needs of each life stage being captured. No included studies examined the effects of interventions on education about global psychosocial functions on cognition in older adults. Yet, half the studies in children were on education interventions, mostly in school-based interventions. This difference is reflected in the outcomes, whereby calculation functions and mental functions of language were more commonly measured in children and adolescents rather than in adults.

Although around half the reviews planned to assess differential effects of interventions across PROGRESS-Plus factors, these analyses were only conducted in eight reviews because of insufficient data. Reviews did not discuss applicability across PROGRESS-Plus factors. There are health inequities in cognitive function across PROGRESS-Plus factors and interventions may have the potential to reduce or exacerbate inequalities [95, 96]. Consequently, it is important to consider equity factors when planning research exploring the effects of life course interventions.

Our assessment of the description of interventions in the reviews showed that only 2/12 items in the TiDieR checklist were adequately reported. Incomplete descriptions of interventions may hinder their effective implementation or replicability. This may be especially relevant when trying to test interventions in different contexts across the world, particularly in low-resource settings.

### Applicability of findings

These findings are limited to reviews of interventions aimed at people without conditions that affect cognitive function and thus are not reflective of the evidence base on treatments for conditions such as dementia.

### Strengths and limitations

This is one of the first reviews to map interventions focusing on cognition, a key health capacity applicable to all people and with outcomes relevant across the life course. Rigorous methods were followed, such as screening, data collection, and quality assessment in duplicate to limit potential biases. We also pre-registered the plan for this study in OSF. We developed a pragmatic approach to mapping reviews of interventions through a life course lens, which can be replicated across different topic areas. Interventions and outcomes were classified into the globally accepted ICHI and ICF frameworks, which provide a detailed coding framework for these concepts. However, these frameworks may not align with other frameworks and other research teams might have adopted a slightly different approach to classifying and grouping interventions and outcomes.

This study is limited by focusing on Cochrane and Campbell reviews, which represent ~15% of reviews published [97], albeit of the highest quality. The findings of this mapping review can be used by both Cochrane and Campbell to inform planning of future reviews on cognition. We are uncertain if these results apply to systematic reviews published in other journals. Another limitation is that we may have missed Cochrane reviews where cognitive function or sleep were secondary outcomes or where the PICO annotation has not yet been completed [98].

## Conclusion

This mapping review summarises existing interventions aimed at promoting cognitive function at different life stages, highlighting key gaps in current evidence. It serves as a starting point to guide future research in identifying what can be done in practice to improve cognition across the life course. The review also identifies key life stages that are insufficiently addressed in existing evidence, aligning with WHO’s framework for implementing a life course approach, which calls for strengthened continuity of service provision across the life course. By mapping cognitive interventions across the life course, this review contributes to WHO’s shift from solely disease-centred approaches to strengthening capacities for health and abilities to experience well-being, recognising that cognition as a key domain of intrinsic capacity for older people, and addressing cognition is part of integrated person-centered care. The variability in outcome measures identified reinforces the need for life course-oriented cognitive measures as appropriate for each life stage.

More systematic reviews and studies adopting the principles of a life course approach, such as promoting health equity and connecting life stages, would help our understanding of cognitive function over time, supporting the translation of evidence to policies and clinical guidelines.

## Supplementary Material

aa-25-0923-File002_afaf306

aa_25_0923_File003_afaf306

## Research Data Transparency and Availability

Data are available in a public, open access repository. The datasets generated and analysed during the current study are available from the OSF repository, DOI: 10.17605/OSF.IO/DS34P.
