# Correction to: Campbell and Cochrane evidence on promoting cognitive capacity across life course: a mapping review

**DOI:** 10.1093/ageing/afag019

**Published:** 2026-02-05

This is a correction to: Vivian A Welch, Monserrat Conde, Victoria I Barbeau, Elizabeth Ghogomu, Shawn Kuria, Hind Sabri, Yanfei Li, Kathy Tong, Ali S Abud, Keshvi Vithlani, Vanessa De Rubeis, Chandni M Jacob, Ritu Sadana, Campbell and Cochrane evidence on promoting cognitive capacity across life course: a mapping review, *Age and Ageing*, Volume 54, Issue 10, October 2025, afaf306, https://doi.org/10.1093/ageing/afaf306

In the originally published paper, affiliations were assigned in error. Those assigned to “Chandni M. Jacob^1,4^” are corrected to read: “Chandni M. Jacob^5^,”; and for “Ritu Sadana^1,4^” are corrected to read: “Ritu Sadana^5^”.

