# Supplementary material for: Campbell and Cochrane evidence on promoting cognitive capacity across life course: a mapping review
Source: Age Ageing. 2025 Oct 26;54(10):afaf306. doi: 10.1093/ageing/afaf306 (PMC12554095; doi:10.1093/ageing/afaf306)
Supplement: aa_25_0923_File003_afaf306 [file aa_25_0923_file003_afaf306.docx]

**Campbell and Cochrane evidence on promoting cognitive capacity across life course: a mapping review - Appendix 2-10**

**Table of contents**

[Appendix 2: Interventions and outcomes 2](#_heading=h.30j0zll)

[Appendix 3: AMSTAR 2 checklist 6](#_heading=h.3znysh7)

[Appendix 4: Excluded Studies 11](#_heading=)

[Appendix 5: WHO regions and World Bank income classification 15](#_heading=h.2p2csry)

[Appendix 6: AMSTAR 2 assessment 17](#_heading=h.23ckvvd)

[Appendix 7: Interventions across life stages in high-quality reviews 18](#_heading=h.ihv636)

[Appendix 8: Interventions across life stage 23](#_heading=h.1hmsyys)

[Appendix 9: Matrix of interventions vs outcomes 29](#_heading=h.3tbugp1)

[Appendix 10: Health equity considerations 30](#_heading=h.nmf14n)

#

#

#

#

#

#

#

#

#

#

#

#

#

# Appendix 2: Interventions and outcomes

The included International International Classification of Health Interventions (ICHI) interventions and Classification of Functioning, Disability and Health (ICF) outcomes are defined below (1,2).

**Table S1.** ICHI intervention definitions and examples

| **ICHI classification** | **ICHI definition** | **Notes and examples** |
| --- | --- | --- |
| ATF.PG.ZZ Assisting and leading exercise for global psychosocial functions | Supporting or guiding exercise focusing on functions as they develop over the life span, required to understand and constructively integrate the mental functions that lead to the formation of the personal and interpersonal skills needed to establish reciprocal social interactions, in terms of both meaning and purpose, as they develop over the life span | E.g., aerobic exercise programmes, active academic lessons |
| ATF.PH.ZZ Training of global psychosocial functions | Teaching, enhancing or developing skills - of functions, as they develop over the life span, required to understand and constructively integrate the mental functions that lead to the formation of the personal and interpersonal skills needed to establish reciprocal social interactions, in terms of both meaning and purpose - through practice | E.g., professional development training, cognitive training, communication skills training |
| ATF.PM.ZZ Education about global psychosocial functions | Providing information to improve knowledge about functions, as they develop over the life span, required to understand and constructively integrate the mental functions that lead to the formation of the personal and interpersonal skills needed to establish reciprocal social interactions, in terms of both meaning and purpose | E.g., school curriculum and governance, early learning educational centers |
| ATF.PN.ZZ Advising about global psychosocial functions | Providing advice to encourage a change of or to maintain functions, as they develop over the life span, required to understand and constructively integrate the mental functions that lead to the formation of the personal and interpersonal skills needed to establish reciprocal social interactions, in terms of both meaning and purpose in relation to health (or risks) |  |
| ATF.PP.ZZ Counselling for global psychosocial functions | Providing therapeutic and/or supportive communication in relation to general mental functions, as they develop over the life span, required to understand and constructively integrate the mental functions that lead to the formation of the personal and interpersonal skills needed to establish reciprocal social interactions, in terms of both meaning and purpose | E.g., cognitive behavioural therapy |
| ATF.RB.ZZ Practical support with global psychosocial functions | Providing practical assistance or guidance to the person in relation to general mental functions, as they develop over the life span, required to understand and constructively integrate the mental functions that lead to the formation of the personal and interpersonal skills needed to establish reciprocal social interactions, in terms of both meaning and purpose | E.g., shift work schedules, vitamin supplements, daycare which does not focus on education |
| ATF.RC.ZZ Emotional support for global psychosocial functions | Providing comfort, empathy or motivational support to the person in relation to general mental functions, as they develop over the life span, required to understand and constructively integrate the mental functions that lead to the formation of the personal and interpersonal skills needed to establish reciprocal social interactions, in terms of both meaning and purpose | Support provided by health professionals/social workers  E.g., home visits |
| ATF.RE.ZZ Provision of peer support for global psychosocial functions | Putting in place arrangements for emotional, social and practical assistance to be given by a person/group who possesses experiential knowledge of a specific behaviour or stressor | Support provided by non-professional with shared experience (e.g., through gender, age, health condition, caregiver)  E.g., befriending services, peer support groups |

**Table S2.** ICF outcome definitions and examples

| **ICF classification** | **ICF definition** | **Notes and examples** |
| --- | --- | --- |
| b114 Orientation functions | General mental functions of knowing and ascertaining one's relation to time to place, to self, to others, to objects, and to space. | E.g., spatial orientation |
| b117 Intellectual functions | General mental functions, required to understand and constructively integrate the various mental functions including all cognitive functions and their development over the life span. | E.g., intellectual development, academic performance (overall), cognition (overall), neurodevelopment outcomes, mental development, executive function, digit symbol |
| b122 Global psychosocial functions | General mental functions, as they develop over the life span, required to understand and constructively integrate the mental functions that lead to the formation of the personal skills needed to establish reciprocal social interactions, in terms of both meaning and purpose. | E.g., behaviour outcomes, socioemotional skills, stress, depression, psychosocial development, loneliness, quality of life |
| b134 Sleep functions | General mental functions of periodic, reversible and selective physical and mental disengagement from one's immediate environment accompanied by characteristic physiological changes. | E.g., total sleep, sleepiness, sleep quality, hot flushes (as a measure of sleep disturbances) |
| b140 Attention functions | Specific mental functions of focusing on an external stimulus or internal experience for the required period of time. | E.g., attention (continuous, divided), reaction time (if focus is attention), alertness |
| B144 Memory functions | Specific mental functions of registering and storing information and retrieving it as needed. | E.g., immediate recall, recognition, delayed recall |
| b147 Psychomotor functions | Specific mental functions of control over both motor and psychological events at the body level. | E.g., psychomotor development, sensorimotor speed, reaction time (if focus is on psychomotor functions), motor outcomes |
| b167 Mental functions of language | Specific mental functions of recognizing and using signs, symbols and other components of a language. | E.g., literacy, reading, writing, spelling, language, communication, linguistic |
| b172 Calculation functions | Specific mental functions of determination, approximation and manipulation of mathematical symbols and processes. | E.g., math, numeracy |
| b330 Fluency and rhythm of speech functions | Functions of the production of flow and tempo of speech. | E.g., word fluency, verbal ability, vocabulary |

## References

1. World Health Organisation. WHO-FIC Maintenance Platform. [cited 2024 Apr 30]. International Classification of Health Interventions (ICHI). Available from:<https://icd.who.int/dev11/l-ichi/en>
2. World Health Organization. International classification of functioning, disability and health : ICF [Internet]. World Health Organization; 2001 [cited 2024 Sep 24]. Available from:<https://iris.who.int/handle/10665/42407>

# Appendix 3: AMSTAR 2 checklist

The complete checklist for the AMSTAR 2 critical appraisal tool for systematic reviews can be found below (1).

## 1. Did the research questions and inclusion criteria for the review include the components of PICO?

| For Yes:  • Population  • Intervention  • Comparator group  • Outcome | Optional (recommended)  • Timeframe for follow-up | • Yes  • No |
| --- | --- | --- |

## 2. Did the report of the review contain an explicit statement that the review methods were established prior to the conduct of the review and did the report justify any significant deviations from the protocol?

| For Partial Yes:  The authors state that they had a written protocol or guide that included ALL the following:  • review question(s)  • a search strategy  • inclusion/exclusion criteria  • a risk of bias assessment | For Yes:  As for partial yes, plus the protocol should be registered and should also have specified:  • a meta-analysis/synthesis plan, if appropriate, and  • a plan for investigating causes of heterogeneity  • a plan for investigating causes of heterogeneity | • Yes  • Partial Yes  • No |
| --- | --- | --- |

## 3. Did the review authors explain their selection of the study designs for inclusion in the review?

| For Yes, the review should satisfy ONE of the following:  • Explanation for including only RCTs  • OR Explanation for including only NRSI  • OR Explanation for including both RCTs and NRSI | | • Yes  • No |
| --- | --- | --- |

## 4. Did the review authors use a comprehensive literature search strategy?

| For Partial Yes (all the following):  • searched at least 2 databases (relevant to research question)  • provided key word and/or search strategy  • justified publication restrictions (e.g. language) | For Yes, should also have (all the following):  • searched the reference lists / bibliographies of included studies  • searched trial/study registries  • included/consulted content experts in the field  • where relevant, searched for grey literature  • conducted search within 24 months of completion of the review | • Yes  • Partial Yes  • No |
| --- | --- | --- |

## 5. Did the review authors perform study selection in duplicate?

| For Yes, either ONE of the following:  • at least two reviewers independently agreed on selection of eligible studies and achieved consensus on which studies to include  • OR two reviewers selected a sample of eligible studies and achieved good agreement (at least 80 percent), with the remainder selected by one reviewer. | | • Yes  • No |
| --- | --- | --- |

## 6. Did the review authors perform data extraction in duplicate?

| For Yes, either ONE of the following:  • at least two reviewers achieved consensus on which data to extract from included studies  • OR two reviewers extracted data from a sample of eligible studies and achieved good agreement (at least 80 percent), with the remainder extracted by one reviewer. | | • Yes  • No |
| --- | --- | --- |

## 7. Did the review authors provide a list of excluded studies and justify the exclusions?

| For Partial Yes:  • provided a list of all potentially relevant studies that were read in full-text form but excluded from the review | For Yes, must also have:  • justified the exclusion from the review of each potentially relevant study | • Yes  • Partial Yes  • No |
| --- | --- | --- |

## 8. Did the review authors describe the included studies in adequate detail?

| For Partial Yes (ALL the following):  • described populations  • described interventions  • described comparators  • described outcomes  • described research designs | For Yes, should also have ALL the following:  • described population in detail  • described intervention in detail (including doses where relevant)  • described comparator in detail (including doses where relevant)  • described study’s setting  • timeframe for follow-up | • Yes  • Partial Yes  • No |
| --- | --- | --- |

##

## 9. Did the review authors use a satisfactory technique for assessing the risk of bias (RoB) in individual studies that were included in the review?

| A. RCTs | | |
| --- | --- | --- |
| For Partial Yes, must have assessed RoB from:  • unconcealed allocation, and  • lack of blinding of patients and assessors when assessing outcomes (unnecessary for objective outcomes such as all-cause mortality) | For Yes, must also have assessed RoB from:  • allocation sequence that was not truly random, and  • selection of the reported result from among multiple measurements or analyses of a specified outcome | • Yes  • Partial Yes  • No  • Includes only NRSI |
| B. NRSI | | |
| For Partial Yes, must have assessed RoB from:  • from confounding, and  • from selection bias | For Yes, must also have assessed RoB:  • methods used to ascertain exposures and outcomes, and  • selection of the reported result from among multiple measurements or analyses of a specified outcome | • Yes  • Partial Yes  • No  • Includes only RCTs |

## 10. Did the review authors report on the sources of funding for the studies included in the review?

| For Yes:  • Must have reported on the sources of funding for individual studies included in the review. Note: Reporting that the reviewers looked for this information but it was not reported by study authors also qualifies | | • Yes  • No |
| --- | --- | --- |

## 11. If meta-analysis was performed did the review authors use appropriate methods for statistical combination of results?

| A. RCTs | | |
| --- | --- | --- |
| For Yes:  • The authors justified combining the data in a meta-analysis  • AND they used an appropriate weighted technique to combine study results and adjusted for heterogeneity if present.  • AND investigated the causes of any heterogeneity | | • Yes  • No  • No meta- analysis conducted |
| B. NRSI | | |
| For Yes:  • The authors justified combining the data in a meta-analysis  • AND they used an appropriate weighted technique to combine study results, adjusting for heterogeneity if present  • AND they statistically combined effect estimates from NRSI that were adjusted for confounding, rather than combining raw data, or justified combining raw data when adjusted effect estimates were not available  • AND they reported separate summary estimates for RCTs and NRSI separately when both were included in the review | | • Yes  • No  • No meta- analysis conducted |

## 12. If meta-analysis was performed, did the review authors assess the potential impact of RoB in individual studies on the results of the meta-analysis or other evidence synthesis?

| For Yes:  • included only low risk of bias RCTs  • OR, if the pooled estimate was based on RCTs and/or NRSI at variable RoB, the authors performed analyses to investigate possible impact of RoB on summary estimates of effect. | | • Yes  • No |
| --- | --- | --- |

## 13. Did the review authors account for RoB in individual studies when interpreting/ discussing the results of the review?

| For Yes:  • included only low risk of bias RCTs  • OR, if RCTs with moderate or high RoB, or NRSI were included the review provided a discussion of the likely impact of RoB on the results | | • Yes  • No |
| --- | --- | --- |

## 14. Did the review authors provide a satisfactory explanation for, and discussion of, any heterogeneity observed in the results of the review?

| For Yes:  • There was no significant heterogeneity in the results  • OR if heterogeneity was present the authors performed an investigation of sources of any heterogeneity in the results and discussed the impact of this on the results of the review | | • Yes  • No |
| --- | --- | --- |

## 15. If they performed quantitative synthesis did the review authors carry out an adequate investigation of publication bias (small study bias) and discuss its likely impact on the results of the review?

| For Yes:  • performed graphical or statistical tests for publication bias and discussed the likelihood and magnitude of impact of publication bias | | • Yes  • No  • No meta- analysis conducted |
| --- | --- | --- |

## 16. Did the review authors report any potential sources of conflict of interest, including any funding they received for conducting the review?

| For Yes:  • The authors reported no competing interests OR  • The authors described their funding sources and how they managed potential conflicts of interest | | • Yes  • No |
| --- | --- | --- |

## References

1. Shea BJ, Reeves BC, Wells G *et al*. AMSTAR 2: a critical appraisal tool for systematic reviews that include randomised or non-randomised studies of healthcare interventions, or both. BMJ 2017; j4008.

# Appendix 4: Excluded Studies

The references and reasons for excluding the 23 reviews that were excluded during full-text screening can be found below.

**Table S3.** Characteristics of excluded studies.

| **Study ID** | **Reason for exclusion** |
| --- | --- |
| Areosa Sastre 2017 [(1)](https://www.zotero.org/google-docs/?upOilX) | Intervention does not aim to improve, maintain or prevent cognitive decline |
| Barlow 2015 [(2)](https://www.zotero.org/google-docs/?Zu9BRc) | Outcomes did not include measures of cognitive capacity |
| Bondebjerg 2023 [(3)](https://www.zotero.org/google-docs/?Y7amhZ) | Population not eligible |
| Brown 2019 [(4)](https://www.zotero.org/google-docs/?FWBJsR) | Outcomes did not include measures of cognitive capacity |
| Centeno 2019 [(5)](https://www.zotero.org/google-docs/?Olqpay) | Outcomes did not include measures of cognitive capacity |
| Dalgaard 2022 [(6)](https://www.zotero.org/google-docs/?WeSbBC) | Does not aim to improve, prevent decline or maintain cognitive function |
| Dalgaard 2022b [(7)](https://www.zotero.org/google-docs/?D6WY70) | Population not eligible |
| dasNair 2016 [(8)](https://www.zotero.org/google-docs/?Zgxr70) | Population not eligible |
| Filges 2015 [(9)](https://www.zotero.org/google-docs/?O0owGC) | Outcomes did not include measures of cognitive capacity |
| Flemming 2019 [(10)](https://www.zotero.org/google-docs/?ivL3nK) | Population not eligible |
| Hafdi 2021 [(11)](https://www.zotero.org/google-docs/?GJvmtb) | Population not eligible |
| Harrison 2022 [(12)](https://www.zotero.org/google-docs/?bc71uy) | Not a systematic review (overview of reviews) |
| Kirkman 2022 [(13)](https://www.zotero.org/google-docs/?ax53vp) | Population not eligible |
| Laermans 2023 [(14)](https://www.zotero.org/google-docs/?O0f2TK) | Outcomes did not include measures of cognitive capacity |
| Lassi 2021 [(15)](https://www.zotero.org/google-docs/?DxKklH) | Outcomes did not include measures of cognitive capacity |
| Littell 2021 [(16)](https://www.zotero.org/google-docs/?Ps5GSG) | Outcomes did not include measures of cognitive capacity |
| Littell 2023 [(17)](https://www.zotero.org/google-docs/?QMlWx6) | Population not eligible |
| Mansfield 2024 [(18)](https://www.zotero.org/google-docs/?D3Ywor) | Outcomes did not include measures of cognitive capacity |
| Piromchai 2015 [(19)](https://www.zotero.org/google-docs/?hi7GEy) | Outcomes did not include measures of cognitive capacity |
| Taylor 2021 [(20)](https://www.zotero.org/google-docs/?UrIW20) | Population not eligible |
| Taylor-Rowan 2023 [(21)](https://www.zotero.org/google-docs/?uRuRoQ) | Intervention does not aim to improve, maintain or prevent cognitive decline |
| Valdebenito 2018 [(22)](https://www.zotero.org/google-docs/?uhw4UC) | Does not aim to improve, prevent decline or maintain cognitive function |
| Yang 2016 [(23)](https://www.zotero.org/google-docs/?gLVbUT) | Population not eligible |

##

##

##

## References

[1. Areosa Sastre A, Vernooij RW, González-Colaço Harmand M, Martínez G. Effect of the treatment of Type 2 diabetes mellitus on the development of cognitive impairment and dementia. Cochrane Dementia and Cognitive Improvement Group, editor. Cochrane Database of Systematic Reviews [Internet]. 2017;2017(6). Available from: http://doi.wiley.com/10.1002/14651858.CD003804.pub2](https://www.zotero.org/google-docs/?l9gpoY)

[2. Barlow J, Bennett C, Midgley N, Larkin SK, Wei Y. Parent‐infant Psychotherapy for Improving Parental and Infant Mental Health: A Systematic Review. Campbell Systematic Reviews [Internet]. 2015 Jan;11(1):1–223. Available from: https://onlinelibrary.wiley.com/doi/10.4073/csr.2015.6](https://www.zotero.org/google-docs/?l9gpoY)

[3. Bondebjerg A, Dalgaard NT, Filges T, Viinholt BCA. The effects of small class sizes on students’ academic achievement, socioemotional development and well‐being in special education: A systematic review. Campbell Systematic Reviews [Internet]. 2023 Sep;19(3):e1345. Available from: https://onlinelibrary.wiley.com/doi/10.1002/cl2.1345](https://www.zotero.org/google-docs/?l9gpoY)

[4. Brown JVE, Walsh V, McGuire W. Formula versus maternal breast milk for feeding preterm or low birth weight infants. Cochrane Neonatal Group, editor. Cochrane Database of Systematic Reviews [Internet]. 2019 Aug 27; Available from: https://doi.wiley.com/10.1002/14651858.CD002972.pub3](https://www.zotero.org/google-docs/?l9gpoY)

[5. Centeno Tablante E, Pachón H, Guetterman HM, Finkelstein JL. Fortification of wheat and maize flour with folic acid for population health outcomes. Cochrane Public Health Group, editor. Cochrane Database of Systematic Reviews [Internet]. 2019 Jul 1;2019(7). Available from: http://doi.wiley.com/10.1002/14651858.CD012150.pub2](https://www.zotero.org/google-docs/?l9gpoY)

[6. Dalgaard NT, Bondebjerg A, Klokker R, Viinholt BCA, Dietrichson J. Adult/child ratio and group size in early childhood education or care to promote the development of children aged 0–5 years: A systematic review. Campbell Systematic Reviews [Internet]. 2022 Jun;18(2):e1239. Available from: https://onlinelibrary.wiley.com/doi/10.1002/cl2.1239](https://www.zotero.org/google-docs/?l9gpoY)

[7. Dalgaard NT, Bondebjerg A, Viinholt BCA, Filges T. The effects of inclusion on academic achievement, socioemotional development and wellbeing of children with special educational needs. Campbell Systematic Reviews [Internet]. 2022 Dec;18(4):e1291. Available from: https://onlinelibrary.wiley.com/doi/10.1002/cl2.1291](https://www.zotero.org/google-docs/?l9gpoY)

[8. Das Nair R, Cogger H, Worthington E, Lincoln NB. Cognitive rehabilitation for memory deficits after stroke. Cochrane Stroke Group, editor. Cochrane Database of Systematic Reviews [Internet]. 2016 Sep 1;2016(9). Available from: http://doi.wiley.com/10.1002/14651858.CD002293.pub3](https://www.zotero.org/google-docs/?l9gpoY)

[9. Filges T, Rasmussen PS, Andersen D, Jørgensen AK. Multidimensional Family Therapy (MDFT) for Young People in Treatment for Non‐opioid Drug Abuse: A Systematic Review. Campbell Systematic Reviews [Internet]. 2015 Jan;11(1):1–124. Available from: https://onlinelibrary.wiley.com/doi/10.4073/csr.2015.8](https://www.zotero.org/google-docs/?l9gpoY)

[10. Fleming P, McGilloway S, Hernon M, Furlong M, O’Doherty S, Keogh F, et al. Individualized funding interventions to improve health and social care outcomes for people with a disability: A mixed‐methods systematic review. Campbell Systematic Reviews [Internet]. 2019 Jun;15(1–2):e1008. Available from: https://onlinelibrary.wiley.com/doi/10.4073/csr.2019.3](https://www.zotero.org/google-docs/?l9gpoY)

[11. Hafdi M, Hoevenaar-Blom MP, Richard E. Multi-domain interventions for the prevention of dementia and cognitive decline. Cochrane Dementia and Cognitive Improvement Group, editor. Cochrane Database of Systematic Reviews [Internet]. 2021 Nov 8;2021(11). Available from: http://doi.wiley.com/10.1002/14651858.CD013572.pub2](https://www.zotero.org/google-docs/?l9gpoY)

[12. Harrison L, Sharma N, Irfan O, Zaman M, Vaivada T, Bhutta ZA. Mental Health and Positive Development Prevention Interventions: Overview of Systematic Reviews. Pediatrics [Internet]. 2022 May 1;149(Supplement 6):e2021053852G. Available from: https://publications.aap.org/pediatrics/article/149/Supplement%206/e2021053852G/186940/Mental-Health-and-Positive-Development-Prevention](https://www.zotero.org/google-docs/?l9gpoY)

[13. Kirkman MA, Day J, Gehring K, Zienius K, Grosshans D, Taphoorn M, et al. Interventions for preventing and ameliorating cognitive deficits in adults treated with cranial irradiation. Cochrane Gynaecological, Neuro-oncology and Orphan Cancer Group, editor. Cochrane Database of Systematic Reviews [Internet]. 2022 Nov 25;2022(11). Available from: http://doi.wiley.com/10.1002/14651858.CD011335.pub3](https://www.zotero.org/google-docs/?l9gpoY)

[14. Laermans J, Scheers H, Vandekerckhove P, De Buck E. Friendly visiting by a volunteer for reducing loneliness or social isolation in older adults: A systematic review. Campbell Systematic Reviews [Internet]. 2023 Dec;19(4):e1359. Available from: https://onlinelibrary.wiley.com/doi/10.1002/cl2.1359](https://www.zotero.org/google-docs/?l9gpoY)

[15. Lassi ZS, Padhani ZA, Rabbani A, Rind F, Salam RA, Bhutta ZA. Effects of nutritional interventions during pregnancy on birth, child health and development outcomes: A systematic review of evidence from low‐ and middle‐income countries. Campbell Systematic Reviews [Internet]. 2021 Jun;17(2):e1150. Available from: https://onlinelibrary.wiley.com/doi/10.1002/cl2.1150](https://www.zotero.org/google-docs/?l9gpoY)

[16. Littell JH, Pigott TD, Nilsen KH, Green SJ, Montgomery OLK. Multisystemic Therapy® for social, emotional, and behavioural problems in youth age 10 to 17: An updated systematic review and meta‐analysis. Campbell Systematic Reviews [Internet]. 2021 Dec;17(4):e1158. Available from: https://onlinelibrary.wiley.com/doi/10.1002/cl2.1158](https://www.zotero.org/google-docs/?l9gpoY)

[17. Littell JH, Pigott TD, Nilsen KH, Roberts J, Labrum TK. Functional Family Therapy for families of youth (age 11–18) with behaviour problems: A systematic review and meta‐analysis. Campbell Systematic Reviews [Internet]. 2023 Sep;19(3):e1324. Available from: https://onlinelibrary.wiley.com/doi/10.1002/cl2.1324](https://www.zotero.org/google-docs/?l9gpoY)

[18. Mansfield L, Daykin N, O’Connell NE, Bailey D, Forde L, Smith R, et al. A mixed methods systematic review on the effects of arts interventions for children and young people at‐risk of offending, or who have offended on behavioural, psychosocial, cognitive and offending outcomes: A systematic review. Campbell Systematic Reviews [Internet]. 2024 Mar;20(1):e1377. Available from: https://onlinelibrary.wiley.com/doi/10.1002/cl2.1377](https://www.zotero.org/google-docs/?l9gpoY)

[19. Piromchai P, Avery A, Laopaiboon M, Kennedy G, O’Leary S. Virtual reality training for improving the skills needed for performing surgery of the ear, nose or throat. Cochrane ENT Group, editor. Cochrane Database of Systematic Reviews [Internet]. 2015 Sep 9;2015(11). Available from: http://doi.wiley.com/10.1002/14651858.CD010198.pub2](https://www.zotero.org/google-docs/?l9gpoY)

[20. Taylor LA, Mhizha-Murira JR, Smith L, Potter KJ, Wong D, Evangelou N, et al. Memory rehabilitation for people with multiple sclerosis. Cochrane Multiple Sclerosis and Rare Diseases of the CNS Group, editor. Cochrane Database of Systematic Reviews [Internet]. 2021 Oct 18;2021(10). Available from: http://doi.wiley.com/10.1002/14651858.CD008754.pub4](https://www.zotero.org/google-docs/?l9gpoY)

[21. Taylor-Rowan M, Alharthi AA, Noel-Storr AH, Myint PK, Stewart C, McCleery J, et al. Anticholinergic deprescribing interventions for reducing risk of cognitive decline or dementia in older adults with and without prior cognitive impairment. Cochrane Dementia and Cognitive Improvement Group, editor. Cochrane Database of Systematic Reviews [Internet]. 2023 Dec 8;2023(12). Available from: http://doi.wiley.com/10.1002/14651858.CD015405.pub2](https://www.zotero.org/google-docs/?l9gpoY)

[22. Valdebenito S, Eisner M, Farrington DP, Ttofi MM, Sutherland A. School‐based interventions for reducing disciplinary school exclusion: a systematic review. Campbell Systematic Reviews [Internet]. 2018 Jan;14(1). Available from: https://onlinelibrary.wiley.com/doi/10.4073/csr.2018.1](https://www.zotero.org/google-docs/?l9gpoY)

[23. Yang A, Wu HM, Tang JL, Xu L, Yang M, Liu GJ. Acupuncture for stroke rehabilitation. Cochrane Stroke Group, editor. Cochrane Database of Systematic Reviews [Internet]. 2016 Aug 26;2016(8). Available from: http://doi.wiley.com/10.1002/14651858.CD004131.pub3](https://www.zotero.org/google-docs/?l9gpoY)

# Appendix 5: WHO regions and World Bank income classification

**Table S4.** Country representation across World Bank income classifications (n=65).

| **World Bank income classification*** | **Number of reviews (%)** |
| --- | --- |
| Low-income economies | 8 (12%) |
| Lower middle-income economies | 25 (38%) |
| Upper middle-income economies | 23 (35%) |
| High-income economies | 54 (83%) |
| Not reported | 3 (5%) |

*Reviews with primary studies conducted across multiple economies appear in the table once for each relevant economy.

**Table S5.** Country representation across WHO regions (n=65).

| **WHO region classification**** | **Number of reviews (%)** |
| --- | --- |
| African Region (AFR) | 14 (22%) |
| Region of the Americas (AMR) | 48 (74%) |
| South-East Asian Region (SEAR) | 20 (31%) |
| European Region (EUR) | 50 (77%) |
| Eastern Mediterranean Region (EMR) | 8 (12%) |
| Western Pacific Region (WPR) | 33 (51%) |
| Not reported | 3 (5%) |

**Reviews with primary studies conducted across multiple regions appear in the table once for each relevant region.

Data on all countries where the primary studies occurred was collected. Countries were then categorized using the 2024-2025 World Bank economies and the World Health Organisation regions (1,2). Twenty-nine (45%) reviews included primary studies across multiple economies and 49 (75%) reviews included studies across multiple regions (Table S4, S5). One review did not have any included studies and two reviews did not specify any countries (3-5).

Most reviews included studies conducted in high-income economies (83%), lower middle-income economies (38%), upper middle-income economies (35%) and the least reviews had studies conducted in low-income countries (12%).

Seventy-seven percent of the reviews included at least one study conducted in Europe while 74% had studies conducted in the Americas, and 51% had studies conducted in Western Pacific region. The least proportion of reviews has studies conducted in the Eastern Mediterranean region (12%).

##

## References

1. The World Bank. The World Bank. [cited 2024 Apr 30]. World Bank Country and Lending Groups – World Bank Data Help Desk. Available from:<https://datahelpdesk.worldbank.org/knowledgebase/articles/906519-world-bank-country-and-lending-groups>
2. World Health Organization. World Health Organization. [cited 2024 Apr 30]. Countries. Available from:<https://www.who.int/countries>
3. Maynard B, Farina A, Dell N, Kelly M. Effects of trauma-informed approaches in schools: A systematic review. Campbell Systematic Reviews [Internet]. 2019;15(1–2):e1018. Available from:<https://onlinelibrary.wiley.com/doi/full/10.1002/cl2.1018>
4. Ooi C, Loke S, Yassin Z, Hamid T. Carbohydrates for improving the cognitive performance of independent‐living older adults with normal cognition or mild cognitive impairment. Cochrane Database of Systematic Reviews [Internet]. 2011;(4). Available from:<http://dx.doi.org/10.1002/14651858.CD007220.pub2>
5. Young L, Embleton N, McCormick F, McGuire W. Multinutrient fortification of human breast milk for preterm infants following hospital discharge. Cochrane Database of Systematic Reviews [Internet]. 2013;(2). Available from:<http://dx.doi.org/10.1002/14651858.CD004866.pub4>

# **Appendix** 6**: AMSTAR** **2 assessment**

The results from the methodological assessment of the 65 included reviews using AMSTAR-2 can be found below.


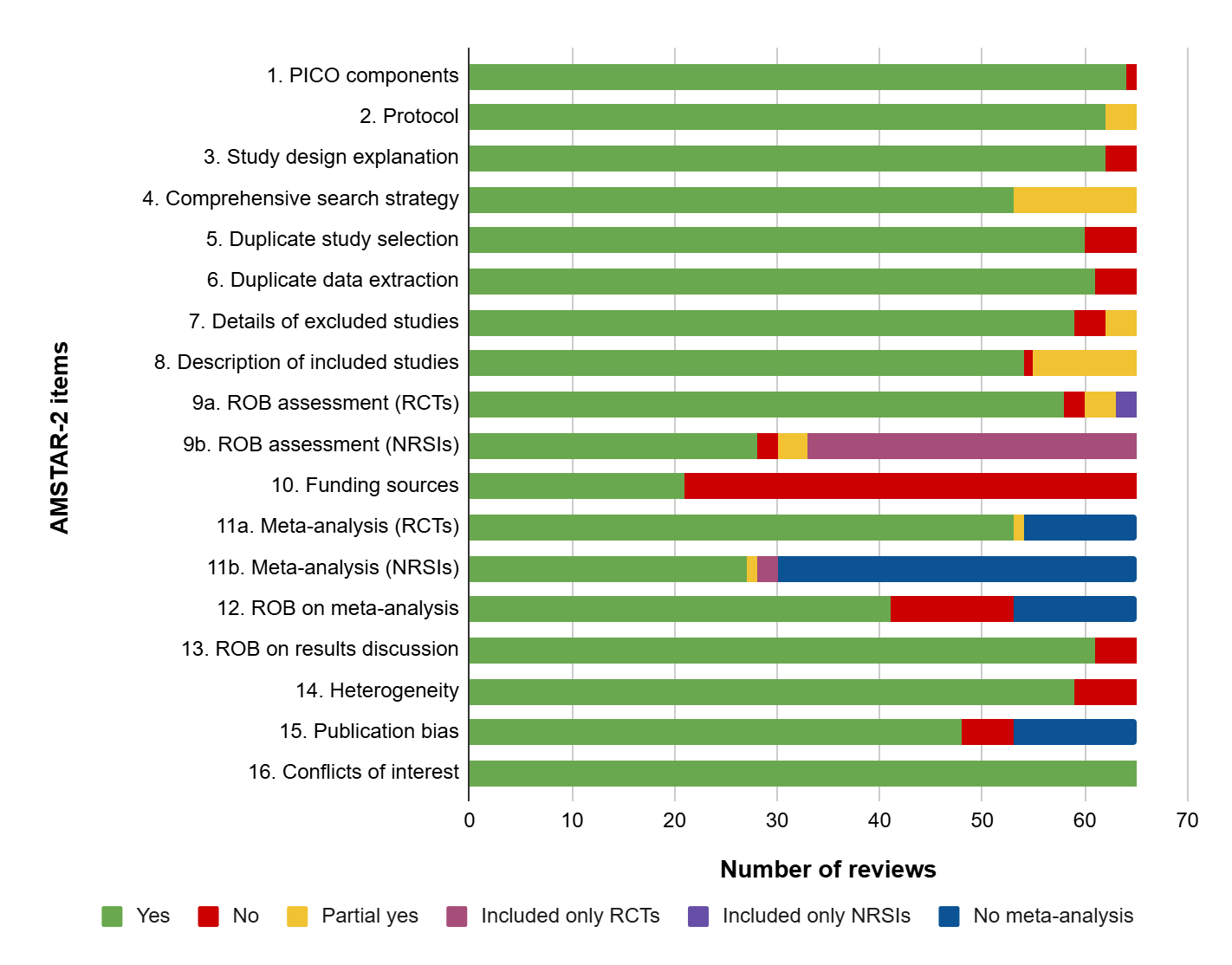


**Figure S1.** Methodological assessment of included reviews (n=65). PICO, population–intervention–control–outcome; ROB, risk of bias; RCT, randomized controlled trial; NRSI, non-randomized studies of intervention.

# Appendix 7: Interventions across life stages in high-quality reviews

**Table S6.** High-quality reviews as classified by their associated intervention(s) and age group(s) (n=35).


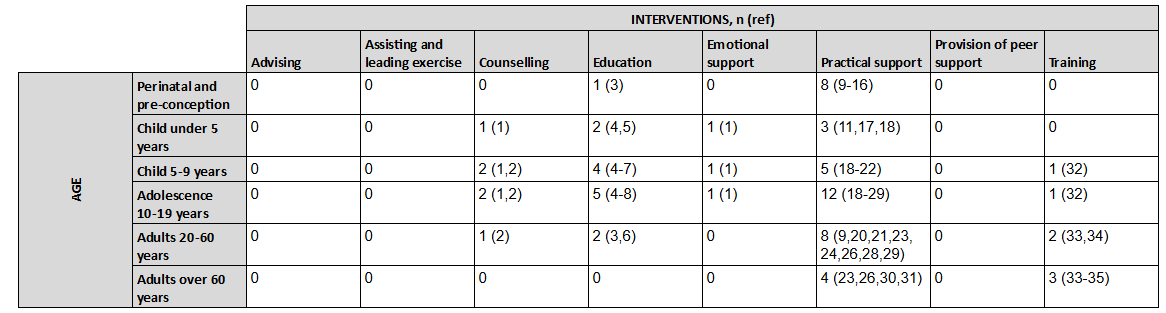


Of the 36 reviews assessed to be high quality using the AMSTAR-2 checklist, 35 could be mapped across the life course by their studied interventions (1-35). The remaining high quality review aimed to assess the practical support intervention of trauma-informed approaches in schools but could not find any relevant studies so no age group was coded (36).

## References

1. Latzman N, Casanueva C, Brinton J, Forman-Hoffman V. The promotion of well-being among children exposed to intimate partner violence: A systematic review of interventions. Campbell Systematic Reviews [Internet]. 2019;15(3). Available from:<https://onlinelibrary.wiley.com/doi/10.1002/cl2.1049>
2. Coren E, Hossain R, Pardo JP, Bakker B. Interventions for promoting reintegration and reducing harmful behaviour and lifestyles in street-connected children and young people: a systematic review. Campbell Systematic Reviews [Internet]. 2016;12(1):1–198. Available from:<https://onlinelibrary.wiley.com/doi/full/10.4073/csr.2016.5>
3. Bryanton J, Beck C, Montelpare W. Postnatal parental education for optimizing infant general health and parent‐infant relationships. Cochrane Database of Systematic Reviews [Internet]. 2013;(11). Available from:<http://dx.doi.org/10.1002/14651858.CD004068.pub4>
4. Randolph J, Bryson A, Menon L, Henderson D, Manuel A, Michaels S, et al. Montessori education’s impact on academic and nonacademic outcomes: A systematic review. Campbell Systematic Reviews [Internet]. 2023;19(3). Available from:<https://onlinelibrary.wiley.com/doi/10.1002/cl2.1330>
5. Filges T, Sonne-Schmidt C, Nielsen B. Small class sizes for improving student achievement in primary and secondary schools: a systematic review. Campbell Systematic Reviews [Internet]. 2018;14(1). Available from:<https://onlinelibrary.wiley.com/doi/10.4073/csr.2018.10>
6. Filges T, Dietrichson J, Viinholt B, Dalgaard N. Service learning for improving academic success in students in grade K to 12: A systematic review. Campbell Systematic Reviews [Internet]. 2022;18(1). Available from:<https://onlinelibrary.wiley.com/doi/10.1002/cl2.1210>
7. Nakamura P, Molotsky A, Zarzur R, Ranjit V, Haddad Y, De Hoop T. Language of instruction in schools in low- and middle-income countries: A systematic review. Campbell Systematic Reviews [Internet]. 2023;12(4). Available from:<https://onlinelibrary.wiley.com/doi/10.1002/cl2.1351>
8. Dietrichson J, Filges T, Klokker R, Viinholt B, Bog M, Jensen U. Targeted school-based interventions for improving reading and mathematics for students with, or at risk of, academic difficulties in Grades 7-12: A systematic review. Campbell Systematic Reviews [Internet]. 2020;16(2). Available from:<https://onlinelibrary.wiley.com/doi/10.1002/cl2.1081>
9. Finkelstein J, Fothergill A, Venkatramanan S, Layden A, Williams J, Crider K, et al. Vitamin B12 supplementation during pregnancy for maternal and child health outcomes. Cochrane Database of Systematic Reviews [Internet]. 2024;(1). Available from:<http://dx.doi.org/10.1002/14651858.CD013823.pub2>
10. Walsh V, Brown J, Askie L, Embleton N, McGuire W. Nutrient‐enriched formula versus standard formula for preterm infants. Cochrane Database of Systematic Reviews [Internet]. 2019;(7). Available from:<http://dx.doi.org/10.1002/14651858.CD004204.pub3>
11. Fenton T, Al-Wassia H, Premji S, Sauve R. Higher versus lower protein intake in formula‐fed low birth weight infants. Cochrane Database of Systematic Reviews [Internet]. 2020;(6). Available from:<http://dx.doi.org/10.1002/14651858.CD003959.pub4>
12. Jasani B, Simmer K, Patole S, Rao S. Long chain polyunsaturated fatty acid supplementation in infants born at term. Cochrane Database of Systematic Reviews [Internet]. 2017;(3). Available from:<http://dx.doi.org/10.1002/14651858.CD000376.pub4>
13. Young L, Embleton N, McGuire W. Nutrient‐enriched formula versus standard formula for preterm infants following hospital discharge. Cochrane Database of Systematic Reviews [Internet]. 2016;(12). Available from:<http://dx.doi.org/10.1002/14651858.CD004696.pub5>
14. Quigley M, Embleton N, McGuire W. Formula versus donor breast milk for feeding preterm or low birth weight infants. Cochrane Database of Systematic Reviews [Internet]. 2019;(7). Available from:<http://dx.doi.org/10.1002/14651858.CD002971.pub5>
15. Moe‐Byrne T, Brown J, McGuire W. Glutamine supplementation to prevent morbidity and mortality in preterm infants. Cochrane Database of Systematic Reviews [Internet]. 2016;(4). Available from:<http://dx.doi.org/10.1002/14651858.CD001457.pub6>
16. Imdad A, Rehman F, Davis E, Ranjit D, Surin G, Attia S, et al. Effects of neonatal nutrition interventions on neonatal mortality and child health and development outcomes: A systematic review. Campbell Systematic Reviews [Internet]. 2021;17(1). Available from:<https://onlinelibrary.wiley.com/doi/10.1002/cl2.1141>
17. Kristjansson B, Petticrew M, MacDonald B, Krasevec J, Janzen L, Greenhalgh T, et al. School feeding for improving the physical and psychosocial health of disadvantaged students. Cochrane Database of Systematic Reviews [Internet]. 2007;(1). Available from:<http://dx.doi.org/10.1002/14651858.CD004676.pub2>
18. Dietrichson J, Filges T, Seerup J, Klokker R, Viinholt B, Bog M, et al. Targeted school-based interventions for improving reading and mathematics for students with or at risk of academic difficulties in Grades K-6: A systematic review. Campbell Systematic Reviews [Internet]. 2021;17(2). Available from:<https://onlinelibrary.wiley.com/doi/10.1002/cl2.1152>
19. Kristjansson B, Petticrew M, MacDonald B, Krasevec J, Janzen L, Greenhalgh T, et al. School feeding for improving the physical and psychosocial health of disadvantaged students. Cochrane Database of Systematic Reviews [Internet]. 2007;(1). Available from:<http://dx.doi.org/10.1002/14651858.CD004676.pub2>
20. Smith T, Thompson A, Maynard B. Self-management interventions for reducing challenging behaviors among school-age students: A systematic review. Campbell Systematic Reviews [Internet]. 2022;18(1). Available from:<https://onlinelibrary.wiley.com/doi/10.1002/cl2.1223>
21. Psaki S, Haberland N, Mensch B, Woyczynski L, Chuang E. Policies and interventions to remove gender-related barriers to girls’ school participation and learning in low- and middle-income countries: A systematic review of the evidence. Campbell Systematic Reviews [Internet]. 2022;18(1). Available from:<https://onlinelibrary.wiley.com/doi/10.1002/cl2.1207>
22. Fisher B, Petrosino A, Persson H, Guckenburg S, Fronius T, Benitez I, et al. School-based law enforcement strategies to reduce crime, increase perceptions of safety, and improve learning outcomes in primary and secondary schools: A systematic review. Campbell Systematic Reviews [Internet]. 2023;19(4). Available from:<https://onlinelibrary.wiley.com/doi/10.1002/cl2.1060>
23. de Vibe M, Bjørndal A, Fattah S, Dyrdal GM, Halland E, Tanner-Smith EE. Mindfulness-based stress reduction (MBSR) for improving health, quality of life and social functioning in adults: a systematic review and meta-analysis. Campbell Systematic Reviews [Internet]. 2017;13(1):1–264. Available from:<https://doi.org/10.4073/csr.2017.11>
24. Hulsegge G, Coenen P, Gascon G, Pahwa M, Greiner B, Bohane C, et al. Adapting shift work schedules for sleep quality, sleep duration, and sleepiness in shift workers. Cochrane Database of Systematic Reviews [Internet]. 2023;(9). Available from:<http://dx.doi.org/10.1002/14651858.CD010639.pub2>
25. Marx R, Tanner‐Smith E, Davison C, Ufholz L, Freeman J, Shankar R, et al. Later school start times for supporting the education, health, and well‐being of high school students. Cochrane Database of Systematic Reviews [Internet]. 2017;(7). Available from:<http://dx.doi.org/10.1002/14651858.CD009467.pub2>
26. Geng J, Dong J, Ni H, Lee M, Wu T, Jiang K, et al. Ginseng for cognition. Cochrane Database of Systematic Reviews [Internet]. 2010;(12). Available from:<http://dx.doi.org/10.1002/14651858.CD007769.pub2>
27. Salam R, Das J, Irfan O, Ahmed W, Sheikh S, Bhutta Z. Effects of preventive nutrition interventions among adolescents on health and nutritional status in low- and middle-income countries: A systematic review. Campbell Systematic Reviews [Internet]. 2020;16(2). Available from:<https://onlinelibrary.wiley.com/doi/10.1002/cl2.1085>
28. Keats E, Oh C, Chau T, Khalifa D, Imdad A, Bhutta Z. Effects of vitamin and mineral supplementation during pregnancy on maternal, birth, child health and development outcomes in low- and middle-income countries: A systematic review. Campbell Systematic Reviews [Internet]. 2021;17(2). Available from:<https://onlinelibrary.wiley.com/doi/10.1002/cl2.1127>
29. Naing C, Whittaker M, Aung H, Chellappan D, Riegelman A. The effects of flipped classrooms to improve learning outcomes in undergraduate health professional education: A systematic review. Campbell Systematic Reviews. 2023;19(3).
30. Rutjes A, Denton D, Di Nisio M, Chong L, Abraham R, Al‐Assaf A, et al. Vitamin and mineral supplementation for maintaining cognitive function in cognitively healthy people in mid and late life. Cochrane Database of Systematic Reviews [Internet]. 2018;(12). Available from:<http://dx.doi.org/10.1002/14651858.CD011906.pub2>
31. Filges T, Siren A, Fridberg T, Nielsen B. Voluntary work for the physical and mental health of older volunteers: A systematic review. Campbell Systematic Reviews [Internet]. 2020;16(4). Available from:<https://onlinelibrary.wiley.com/doi/10.1002/cl2.1124>
32. Turner H, Ncube M, Turner A, Boruch R, Ibekwe N. What are the effects of Teach For America on Math, English Language Arts, and Science outcomes of K-12 students in the USA? Campbell Systematic Reviews [Internet]. 2018;14(1). Available from:<https://onlinelibrary.wiley.com/doi/10.4073/csr.2018.7>
33. Gates N, Rutjes A, Di Nisio M, Karim S, Chong L, March E, et al. Computerised cognitive training for maintaining cognitive function in cognitively healthy people in midlife. Cochrane Database of Systematic Reviews [Internet]. 2019;(3). Available from:<http://dx.doi.org/10.1002/14651858.CD012278.pub2>
34. Saensak S, Vutyavanich T, Somboonporn W, Srisurapanont M. Relaxation for perimenopausal and postmenopausal symptoms. Cochrane Database of Systematic Reviews [Internet]. 2014;(7). Available from:<http://dx.doi.org/10.1002/14651858.CD008582.pub2>
35. Gates N, Rutjes A, Di Nisio M, Karim S, Chong L, March E, et al. Computerised cognitive training for 12 or more weeks for maintaining cognitive function in cognitively healthy people in late life. Cochrane Database of Systematic Reviews [Internet]. 2020;(2). Available from:<http://dx.doi.org/10.1002/14651858.CD012277.pub3>
36. Maynard B, Farina A, Dell N, Kelly M. Effects of trauma-informed approaches in schools: A systematic review. Campbell Systematic Reviews [Internet]. 2019;15(1–2):e1018. Available from:<https://onlinelibrary.wiley.com/doi/full/10.1002/cl2.1018>

# Appendix 8: Interventions across life stage

Breakdown of the interventions evaluated and the outcomes measured in each age group across life span. Additional information on reviews examining exclusively one age group is also provided. The full dataset is available on the Open Science Framework (<https://osf.io/ds34p/>) and can be filtered by age, intervention, outcome, and quality to identify specific studies.

## Perinatal and preconception

**Table S7.** Interventions and outcomes across preconception and perinatal age group (n=13).

| **Number (%) of reviews** | **Interventions** | **Outcome measures** |
| --- | --- | --- |
| 10 (80%) | Practical support with global psychosocial functions | Intellectual functions  Global psychosocial functions  Mental functions of language  Psychomotor functions |
| 1 (8%) | Training of global psychosocial functions | Intellectual functions  Global psychosocial functions |
| 1 (8%) | Education about global psychosocial functions | Sleep |
| 1 (8%) | Education about global psychosocial functions + Practical support with global psychosocial functions | Intellectual functions  Psychomotor functions |

Of the 13 (20%) reviews that included participants in this age group, three (23%) also included other age groups. The reviews assessed different types of interventions, especially practical support with global psychosocial functions, and measured different outcomes.

Eight of the nine reviews that included only preterm or low birth weight infants assessed the impact of practical support with global psychosocial functions interventions (long chain polyunsaturated fatty acid supplementation, glutamine supplementation, formula, fortified breast milk, probiotic supplementation, and taurine supplementation). One review assessed the impact of education about global psychosocial functions + practical support with global psychosocial functions interventions (early developmental programs).

## Children under 5 years

**Table S8.** Interventions and outcomes in children under 5 years (n=17).

| **Number (%) of reviews** | **Interventions** | **Outcome measures** |
| --- | --- | --- |
| 6 (35%) | Education about global psychosocial functions | Intellectual functions; Calculation functions; Mental functions of language; Fluency and rhythm of speech |
| 5 (29%) | Practical support with global psychosocial functions | Intellectual functions; Calculation functions;  Global psychosocial functions; |
| 1 (6%) | Practical support with global psychosocial functions + Education about global psychosocial functions | Mental functions of language |
| 1 (6%) | Training of global psychosocial functions | Intellectual functions; Global psychosocial functions; Attention functions |
| 1 (6%) | Education about global psychosocial functions + Training of global psychosocial functions | Mental functions of language |
| 1 (6%) | Assisting and leading exercise for global psychosocial functions + Practical support with global psychosocial functions + Education about global psychosocial functions | Intellectual functions; Calculation functions; Mental functions of language |
| 1 (6%) | Provision of peer support for global psychosocial functions | Intellectual functions;  Global psychosocial functions |
| 1 (6%) | Emotional support for global psychosocial functions + Counselling for global psychosocial functions | Intellectual functions |

Children under 5 years were included in 17 reviews (27%) and within those reviews, only four of them (24%) had exclusively children under 5 years old.

Of the two reviews with only children under 5 years, one assessed the impact of practical support with global psychosocial functions (feeding) while one assessed the impact of education about global psychosocial functions (Tools of the Mind curriculum).

## Children 5-9 years

**Table S9.** Interventions and outcomes in children 5-9 years (n=25).

| **Number (%) of reviews** | **Interventions** | **Outcome measures** |
| --- | --- | --- |
| 10 (40%) | Education about global psychosocial functions | Intellectual functions; Calculation functions; Mental functions of language; Global psychosocial functions; Fluency and rhythm of speech functions |
| 6 (24%) | Practical support with global psychosocial functions | Intellectual functions; Calculation functions; Mental functions of language; |
| 3 (12%) | Training of global psychosocial functions | Intellectual functions; Calculation functions; Mental functions of language; Global psychosocial functions; Attention functions |
| 1 (4%) | Education about global psychosocial functions + Practical support with global psychosocial functions | Mental functions of language |
| 1 (4%) | Education about global psychosocial functions + Training of global psychosocial functions | Mental functions of language |
| 1 (4%) | Assisting and leading exercise for global psychosocial functions + Education about global psychosocial functions + Practical support with global psychosocial functions | Intellectual functions; Calculation functions; Mental functions of language |
| 1 (4%) | Counselling for global psychosocial functions | Not reported |
| 1 (4%) | Emotional support for global psychosocial functions + Counselling for global psychosocial functions | Intellectual functions |
| 1 (4%) | Provision of peer support for global psychosocial functions | Intellectual functions; Global psychosocial functions |

This age group was covered in 25 reviews (39%), and none were exclusive to this age group. Three reviews planned to measure cognitive capacity function outcomes but did not due to lack of data. The most common intervention type assessed was education about global psychosocial functions and three main outcomes were measured across the reviews including intellectual functions, mental functions of language and calculation functions.

## Adolescents 10-19 years

**Table S10.** Interventions and outcomes in adolescents 10-19 years old (n=34).

| **Number (%) of reviews** | **Interventions** | **Outcome measures** |
| --- | --- | --- |
| 14 (41%) | Practical support with global psychosocial functions | Intellectual functions; Global psychosocial functions; Sleep; Mental functions of language; Calculation functions; Attention functions; Psychomotor functions |
| 9 (26%) | Education about global psychosocial functions | Intellectual functions; Global psychosocial functions; Mental functions of language; Calculation functions; Fluency and rhythm of speech functions |
| 4 (12%) | Training of global psychosocial functions | Intellectual functions; Global psychosocial functions; Mental functions of language; Calculation functions; Attention functions |
| 1 (3%) | Practical support with global psychosocial functions + Education about global psychosocial functions | Mental functions of language |
| 1 (3%) | Education about global psychosocial functions + Training of global psychosocial functions | Mental functions of language |
| 1 (3%) | Counselling for global psychosocial functions | Not reported |
| 1 (3%) | Emotional support for global psychosocial functions | Psychomotor functions |
| 1 (3%) | Assisting and leading exercise for global psychosocial functions + Education about global psychosocial functions + Practical support with global psychosocial functions | Intellectual functions; Mental functions of language; Calculation functions; |
| 1 (3%) | Provision of peer support for global psychosocial functions | Intellectual functions; Global psychosocial functions |
| 1 (3%) | Emotional support for global psychosocial functions + Counselling for global psychosocial functions | Intellectual functions |

Adolescents were included in 34 reviews (53%) in total and within those reviews only three (9%) were exclusive to the age group. The most common interventions were practical support with global psychosocial functions and education about global psychosocial functions and the most common outcome measured was intellectual function.

Two reviews including only adolescents assessed the effects of practical support with global psychosocial functions (later school start times and preventive nutrition interventions, respectively) on sleep and intellectual functions. One review including only adolescents assessed the effect of education about global psychosocial functions (targeted school-based interventions for improving reading and mathematics) on mental functions of language, calculation functions and fluency and rhythm of speech functions.

##

## Adults 20-60 years

**Table S11.** Interventions and outcomes in adults 20-60 years old (n=23).

| **Number (%) of reviews** | **Interventions** | **Outcome measures** |
| --- | --- | --- |
| 14 (61%) | Practical support with global psychosocial functions | Intellectual functions; Global psychosocial functions; Sleep; Mental functions: Calculation functions; Attention functions; Psychomotor functions; Fluency and rhythm of speech functions |
| 3 (13%) | Training of global psychosocial functions | Intellectual functions; Sleep |
| 2 (9%) | Education about global psychosocial functions | Intellectual functions; Global psychosocial functions; Sleep; Mental functions: Calculation functions |
| 1 (4%) | Education about global psychosocial functions + Training of global psychosocial functions | Mental functions of language |
| 1 (4%) | Counselling for global psychosocial functions | Not reported |
| 1 (4%) | Provision of peer support for global psychosocial functions | Intellectual functions; Global psychosocial functions |
| 1 (4%) | Emotional support for global psychosocial functions | Psychomotor functions |

Twenty-three (36%) reviews included adults 20-60 years old and but only two (9%) of them were exclusive. The most common interventions were practical support with global psychosocial functions and different outcomes were assessed in the reviews. One review that assessed counselling for global psychosocial functions planned but did not measure any cognitive capacity function outcomes.

The two reviews that included only adults 20-60 years old assessed the effects of practical support with global psychosocial functions (omega-3 fatty acid addition during pregnancy and sleep interventions) on intellectual functions, mental functions, attention functions, psychomotor functions, and sleep outcomes.

## Older people over 60 years

**Table S12.** Interventions and outcomes in older people over 60 years old (n=15).

| **Number (%) of reviews** | **Interventions** | **Outcome measures** |
| --- | --- | --- |
| 9 (60%) | Practical support with global psychosocial functions | Intellectual functions; Global psychosocial functions; Sleep; Mental functions: Calculation functions; Attention functions; Psychomotor functions; Fluency and rhythm of speech functions |
| 3 (20%) | Training of global psychosocial functions | Intellectual functions; Sleep |
| 1 (7%) | Emotional support for global psychosocial functions | Psychomotor functions |
| 1(7%) | Assisting and leading exercise for global psychosocial functions | Intellectual functions; Attention functions; Psychomotor functions |
| 1(7%) | Provision of peer support for global psychosocial functions | Intellectual functions; Global psychosocial functions |

Fifteen reviews (23%) included this age group and seven (47%) of them were exclusive to this group. The most common intervention was practical support with global psychosocial functions and the most common outcomes were intellectual functions, global psychosocial and psychomotor outcomes.

Of the seven reviews that included only older people, five assessed the effects of practical support with global psychosocial functions on intellectual functions, global psychosocial functions, mental functions, and psychomotor functions. One review assessed the impact of practical support but measured no outcomes. One review assessed the effect of assisting and leading exercise for global psychosocial functions on intellectual functions, attention functions and psychomotor functions.

#

# Appendix 9: Matrix of interventions vs outcomes

Interventions evaluated and outcomes measured were mapped across the reviews (n=65).

#
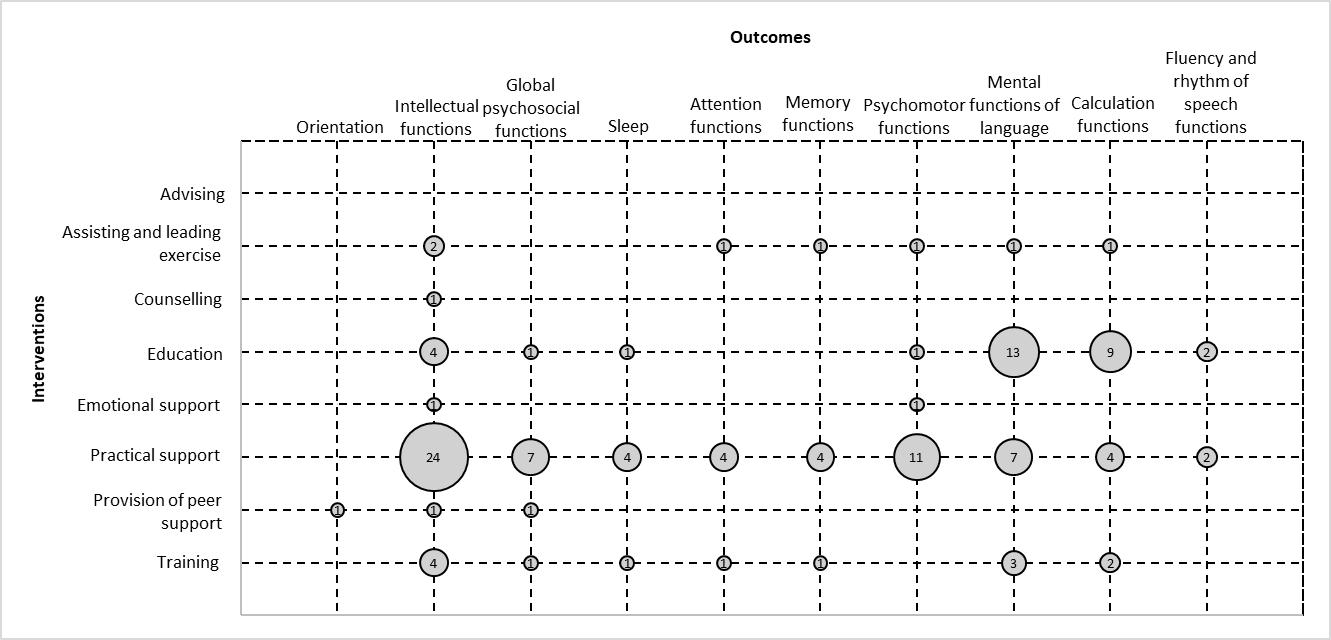


**Figure S2.** Matrix of reviews as classified by their associated intervention(s) and outcome(s). The size of the circles corresponds to the number of reviews (n=65).

# Appendix 10: Health equity considerations

**Table S13.** Health equity considerations described in the reviews (n=65).

| **PROGRESS-Plus Factors** | **Reviews n (%)** | | |
| --- | --- | --- | --- |
|  | **Sociodemographic characteristics** | **Equity analysis: Planned** | **Equity analysis: Actual** |
| Place of Residence (urban/rural) | 17 (26%) | 4 (6%) | 0 (0%) |
| Race/Ethnicity | 23 (35%) | 4 (6%) | 1 (2%) |
| Occupation | 10 (15%) | 2 (3%) | 0 (0%) |
| Gender or Sex | 50 (77%) | 13 (20%) | 2 (3%) |
| Religion | 1 (2%) | 0 (0%) | 0 (0%) |
| Education | 12 (18%) * | 4 (6%) | 4 (6%) |
| Socioeconomic status | 20 (31%) | 16 (25%) | 3 (5%) |
| Social capital (e.g. marital status) | 8 (12%) | 0 (0%) | 0 (0%) |
| Plus factor - Disability | 8 (12%) | 3 (5%) | 1 (2%) |
| Plus factor - Health status (e.g. dementia, disease severity) | 14 (22%) | 4 (6%) | 2 (3%) |
| Plus factor - Age | 64 (98%) | 18 (28%) | 3 (5%) |

*Only parental education was collected for studies focusing on children and adolescents
